# Engagement in primary health care among marginalized people who use drugs in Ottawa, Canada

**DOI:** 10.1186/s12913-020-05670-z

**Published:** 2020-09-07

**Authors:** Claire E. Kendall, Lisa M. Boucher, Jessy Donelle, Alana Martin, Zack Marshall, Rob Boyd, Pam Oickle, Nicola Diliso, Dave Pineau, Brad Renaud, Sean LeBlanc, Mark Tyndall, Ahmed M. Bayoumi

**Affiliations:** 1 Bruyère Research Institute, 43 Bruyère Street, Annex E, Ottawa, Ontario K1N 5C8 Canada; 2 ICES, Ottawa Hospital, Civic Campus, 1053 Carling Avenue, Box 684, Administrative Services Building, 1st Floor, Ottawa, Ontario K1Y 4E9 Canada; 3 Somerset West Community Health Centre, 55 Eccles Street, Ottawa, Ontario K1R 6S3 Canada; 4 PROUD Community Advisory Committee, Ottawa, Ontario Canada; 5 School of Social Work, McGill University, 3506 University Street, Room 421, Montreal, Quebec H3A 2A7 Canada; 6 Sandy Hill Community Health Centre, 221 Nelson Street, Ottawa, Ontario, K1N 1C7 Canada; 7 Ottawa Public Health, 179 Clarence Street, Ottawa, Ontario, K1N 1B3 Canada; 8 Drug Users Advocacy League, Ottawa, Ontario Canada; 9 School of Population and Public Health, University of British Columbia, 2206 East Mall, Vancouver, British Columbia V6T 1Z3 Canada; 10 MAP Centre for Urban Health Solutions, Li Ka Shing Knowledge Institute and Division of General Internal Medicine, St. Michael’s Hospital; Department of Medicine and Institute of Health Policy, Management, and Evaluation, University of Toronto, 30 Bond Street, Toronto, Ontario M5B 1W8 Canada

**Keywords:** People who use drugs, Primary care, Health administrative data

## Abstract

**Background:**

There may be less primary health care engagement among people who use drugs (PWUD) than among the general population, even though the former have greater comorbidity and more frequent use of emergency department care. We investigated factors associated with primary care engagement among PWUD.

**Methods:**

The Participatory Research in Ottawa: Understanding Drugs (PROUD) cohort study meaningfully engaged and trained people with lived experience to recruit and survey marginalized PWUD between March–December 2013. We linked this survey data to provincial-level administrative databases held at ICES. We categorized engagement in primary care over the 2 years prior to survey completion as: not engaged (< 3 outpatient visits to the same family physician) versus engaged in care (3+ visits to the same family physician). We used multivariable logistic regression to determine factors associated with engagement in primary care.

**Results:**

Characteristics of 663 participants included a median age of 43 years, 76% men, and 67% living in the two lowest income quintile neighborhoods. Despite high comorbidity and a median of 4 (interquartile range 0–10) primary care visits in the year prior to survey completion, only 372 (56.1%) were engaged in primary care. Engagement was most strongly associated with the following factors: receiving provincial benefits, including disability payments (adjusted odds ratio [AOR] 4.14 (95% confidence interval [CI] 2.30 to 7.43)) or income assistance (AOR 3.69 (95% CI 2.00 to 6.81)), having ever taken methadone (AOR 3.82 (95% CI 2.28 to 6.41)), mental health comorbidity (AOR 3.43 (95% CI 2.19 to 5.38)), and having stable housing (AOR 2.09 (95% CI 1.29 to 3.38)).

**Conclusions:**

Despite high comorbidity, engagement in primary care among PWUD was low. Our findings suggest that social care (housing, disability, and income support) and mental health care are associated with improved primary care continuity; integration of these care systems with primary care and opioid substitution therapy may lessen the significant morbidity and acute care use among PWUD.

## Background

People who use drugs (PWUD) experience disproportionately high comorbidity and disability [1, 2] as well as excess and premature mortality [3, 4]. Common comorbidities among PWUD include mental health conditions, HIV and hepatitis C [2, 5, 6]. Despite this high burden of illness, the large majority of people who use drugs have unmet health needs [7–9]. In turn, PWUD have disproportionately greater use of acute care services, including emergency department visits and hospital admissions for issues including mental health and substance use diagnoses, and also infectious complications such as soft tissue infections and pneumonia [2, 10, 11].

There is strong evidence that care for complex populations is most effectively and equitably provided in well-supported primary care settings [12–15]. In contrast to other populations, where frequent emergency department visits correlate with poor access to primary care, PWUD typically have both high emergency department visit rates and frequent contacts with primary care providers [2, 10, 16]. However, frequency of contact may not be the best measure of primary care engagement, particularly if individuals do not have continuous access to the *same* doctor over time. For example, we have found that for PWUD, compared to not having a regular family physician, having a regular family physician reduces the odds of having multiple emergency department visits by 50% [2]. This is consistent with a broad literature evidence base demonstrating patient-reported health outcomes and health system improvements associated with having a regular source of primary care, especially for complex populations [17–19].

The objective of this study was to describe regular engagement in primary care and variables associated with this engagement among a cohort of PWUD in Ontario, Canada. We hypothesized that many PWUD would not be receiving regular care from one family physician and that multiple factors would be associated with having a regular family physician [20]. We used data from the Participatory Research in Ottawa: Understanding Drugs (PROUD) study [21], a community-based study of marginalized PWUD in Ottawa who participated in survey design, administration, and analysis. We linked PROUD data to provincial administrative databases, creating a unique dataset with rich individual-level information about health services use within a single payer system with universal access to physician services.

## Methods

### Theoretical influences

To define our variable selection, we were guided by the Rhodes’ ‘Risk Environment Framework’ [20, 22]. Focusing on the HIV risk environment among PWUD, this framework was used in designing the PROUD study protocol, including creation of the survey tool [21]. It was also incorporated in previous analysis of the survey data [23], as well as in a qualitative sub-study conducted by the PROUD team [24]. In the present study, our selection process with the study’s Community Advisory Committee aimed to include variables that represent several different risk environments (i.e. social, economic, physical, policy).

### Data sources

PROUD cohort study: As described previously [21], the PROUD study used street-based peer recruitment and a snowball sampling approach to enrol participants who completed a cross-sectional survey. Our focus was on socially and economically marginalized PWUD. Eligibility criteria included being 16 years of age or older and reporting having injected or smoked drugs other than marijuana in the 12 months prior to enrolment (March to December 2013). Participants completed a peer- or medical student-administered survey that included questions about socio-demographic information, substance use, environmental-structural factors (e.g., legal issues, housing), interpersonal relationships (e.g., connection to community, sexual history), harm reduction practices, health status, and health and social services use. All PROUD study activities were governed by a Community Advisory Committee of PWUD and allies.

ICES databases: For consenting PROUD participants, additional data were obtained by linking the survey responses with health administrative databases held at ICES (www.ices.on.ca). ICES datasets use unique encoded identifiers and are analyzed at ICES. We linked data from PROUD participants deterministically using these identifiers based on participants’ reported Ontario Health Insurance Plan (OHIP) numbers if available, or probabilistically based on their names, dates of birth, and postal codes. We identified participants with duplicate enrolment following linkage and retained responses with the most complete data.

We used the following ICES databases for our study: the Registered Persons database, to obtain demographic data for all residents eligible for provincial health care; the Discharge Abstract Database, to identify all provincial hospital admission and discharge data; the National Ambulatory Care Reporting System, to obtain encounter-level information on visits to emergency departments, including discharge diagnoses; the 2006 Statistics Canada Census data, to estimate socioeconomic status by attributing an income quintile by linking postal code of residence to the mean household income by dissemination area, which represents a standard geographic area typically consisting of 400 to 700 individuals; and the Ontario Drug Benefits, to identify prescription claims by individuals age 65 or older or those receiving income assistance (Ontario Works), disability payments (Ontario Disability Support Program), or provincially-subsidized catastrophic drug coverage (Trillium). We included three databases to capture the distinct ways in which primary care is delivered in Ontario: the OHIP billing claims system, which captures fee-for-service physician services provided in the province; the Community Health Centre database, to identify encounter information for patients seen in Ontario’s Community Health Centres; and the Client Agency Program Enrolment Registry, which compiles encounters for patients who are rostered to family physicians.

### Variables

In general, we used administrative data for variables associated with health care and medication use and most diagnoses and PROUD survey data for other variables, including those that are not captured in administrative records. We categorized gender using self-reported gender in the PROUD survey except when gender was missing or when participants reported gender as “two-spirited” or “other”, in which case we used ICES data (sex at birth). We excluded transgender individuals due to the risk of re-identification (< 6 participants). We used postal code to assign neighbourhood income into quintiles. We classified comorbidity using the Johns Hopkins Adjusted Clinical Groups Case-Mix Assignment software (Sun Microsystems Inc., Santa Clara, CA) by assigning up to 32 distinct Aggregated Diagnosis Groups (ADGs) based on condition duration, severity, diagnostic certainty, etiology, and specialty care involvement (www.hopkinsacg.org) [25]. We categorized comorbidity as low (≤5 ADGs), medium (6–9 ADGs), or high (≥10 ADGs). We used validated ICES algorithms to classify the prevalence of mental health conditions and HIV, and have included these algorithms in Appendix in Table 4 [26, 27]. We calculated primary care visits from the OHIP and Community Health Centre databases.

### Outcomes

Our primary outcome was engagement in primary care, which we defined as 3 or more visits to the same family physician in the 2 years prior to PROUD survey completion [28]. For this assignment, we excluded visits that physician billing claims indicated were primarily for opioid substitution therapy, since in our setting such visits are not usually directed towards comprehensive primary care (we did include such visits in a sensitivity analysis). Engagement was categorized irrespective of the model of primary care; that is, if a patient was contractually rostered to one family physician but had at least 3 visits and the majority of their primary care visits elsewhere, they would be assigned as engaged in care with the physician they saw most frequently.

### Analyses

We used descriptive statistics to summarize our cohort, stratified by primary care engagement category, including measures of central tendencies and dispersion. We compared engagement status using Wilcoxon rank sum tests for continuous variables and chi squared tests or Fisher’s exact test as appropriate for categorical variables. We used logistic regression to analyze variables associated with primary care engagement. We used a non-parsimonious approach and Community Advisory Committee input to selecting covariates but excluded those that we judged likely to be collinear to avoid overfitting the model (e.g. we removed “Detained in jail overnight or longer *ever*” but kept “Detained in jail overnight or longer *in the last 12 months*”). We removed primary care visits from our multivariable model as they are directly associated with the outcome (number of care encounters is used to determine primary care engagement status). We reported associations as odds ratios with 95% confidence intervals.

For several PROUD variables, participants had response options of “no answer” or “don’t know/unsure”. Other variables had missing values. Our primary analyses used a complete case approach; we also conducted a sensitivity analysis in which questions with missing, “don’t know/unsure”, and “no answer” response categories were dichotomized (yes versus no), with the no category including any non-yes response.

We used a *p*-value threshold of 0.05 to determine statistical significance. Cell sizes of 6 or less were reported in aggregate to preserve confidentiality. SAS statistical software version 9.4 (SAS Institute Inc., Cary, N.C.) was used to conduct all statistical analyses.

This study received approval from the Ottawa Health Sciences Network Research Ethics Board (OHSN-REB #20120566-01H). The use of administrative data in this project was authorized under section 45 of Ontario’s *Personal Health Information Protection Act*, which does not require review by a Research Ethics Board.

## Results

**Fig. 1:**
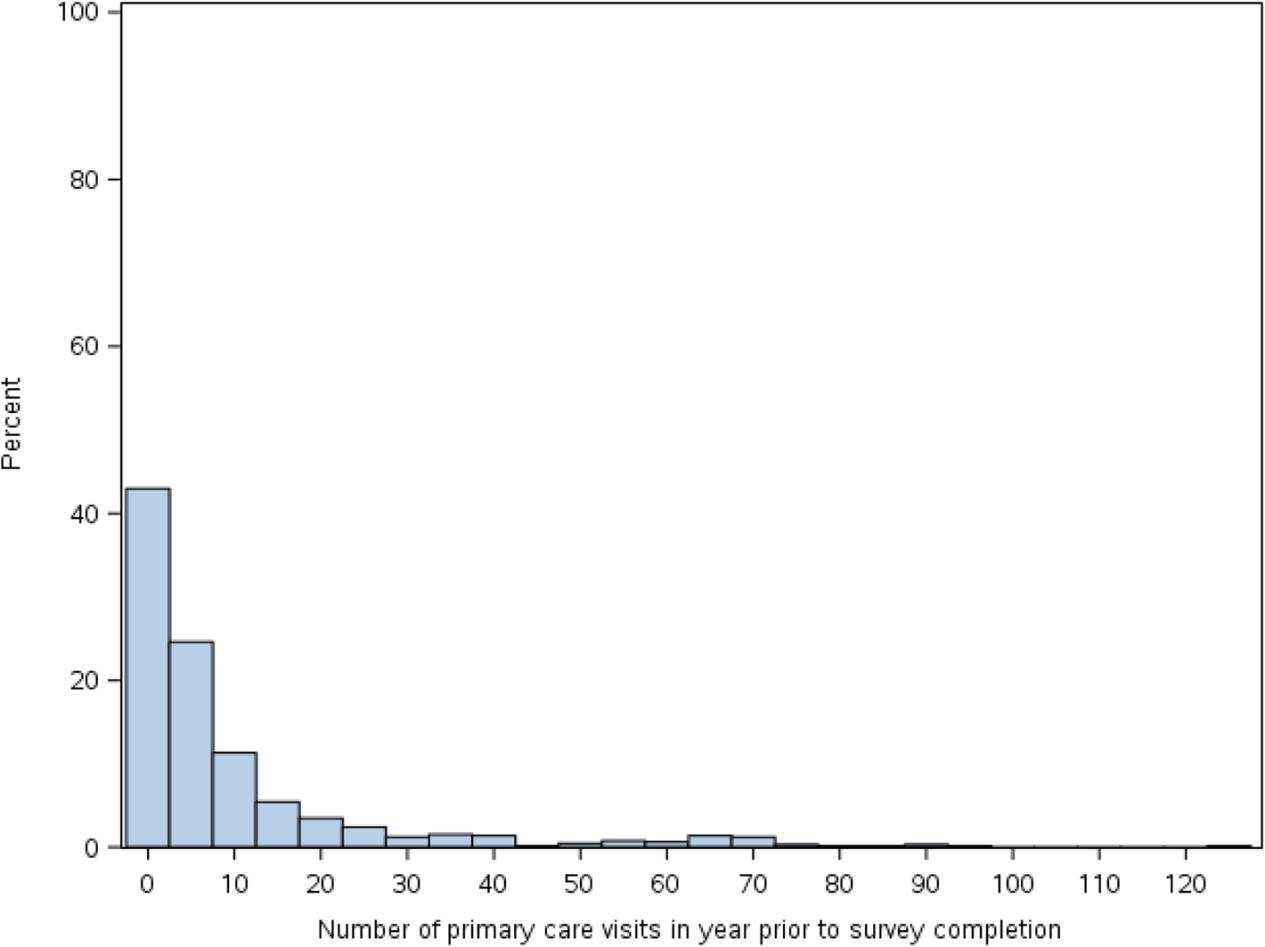
Distribution of primary care visits in year prior to survey completion

Between March and December 2013, 858 PROUD participants completed the survey. Of these, 798 participants agreed to linkage to ICES, and, after we excluded duplicate enrolments and those who did not have Ontario health insurance, 663 of 782 participants (85%) were successfully linked. Among linked participants, 76% were men, the median age was 43 years, 67% lived in a neighbourhood in one of the two lowest income quintiles, and 76% received either disability or income assistance. Table 1 highlights further characteristics from both administrative and self-reported data. By self-report, 56.2% had a regular doctor and 60.2% reported seeking care in a health clinic, doctor’s office or walk-in clinic in the previous 12 months. By administrative data, participants had a median of 4 primary care visits (interquartile range [IQR] 0–10) in the year prior to survey completion (Fig. 1). In the year prior to survey completion, 18.3% had no primary care visits captured in administrative data.

**Table 1:** Baseline Characteristics of PROUD participants

Variable	*N* = 663
**Demographic characteristics**	**n (%)**
Age	
Mean (SD)	41.4 (10.8)
Median (IQR)	43 (33–50)
Age category (years)	
< = 24	54 (8.1%)
25 to 34	134 (20.2%)
35 to 44	182 (27.5%)
45+	293 (44.2%)
Gender	
Male	501 (75.6%)
Female	162 (24.4%)
Ethnicity	
Indigenous only^a^	120 (18.1%)
Other/no answer	543 (81.9%)
First language	
French	102 (15.4%)
English	512 (77.2%)
Other/no answer	49 (7.4%)
Sexual orientation	
Heterosexual	587 (88.5%)
Gay/lesbian/homosexual/other	76 (11.5%)
Neighborhood of residence	
Market/Lowertown	273 (41.2%)
Centretown	117 (17.6%)
Other	273 (41.2%)
Neighborhood Income Quintile	
1 (lowest)	246 (37.1%)
2	196 (29.6%)
3	144 (21.7%)
4 and 5 (highest)	64 (9.6%)
Missing	13 (2.0%)
Highest level of education	
Some high school or less	308 (46.5%)
High school graduate or equivalent	193 (29.1%)
Some college or university	99 (14.9%)
College or university completed	63 (9.5%)
Provincial social assistance benefits	
Disability payments (Ontario Disability Support Program)	342 (51.6%)
Income assistance (Ontario Works)	164 (24.7%)
Other (includes Trillium, 65y+, none)	157 (23.7%)
Comorbidity	
No comorbidity (0 ADGs)^b^	82 (12.4%)
Low comorbidity (1–5 ADGs)	242 (36.5%)
Medium comorbidity (6–9 ADGs)	169 (25.5%)
High comorbidity (> = 10 ADGs)	170 (25.6%)
**Social characteristics**	**n (%)**
Received drugs, money, gifts for sex last 12 months	82 (12.4%)
Stable housing	248 (37.4%)
Ever red zoned	212 (32.0%)
Detained in jail overnight or longer ever	510 (76.9%)
Detained in jail overnight or longer last 12 months	252 (38.0%)
**Drug use characteristics**	**n (%)**
Ever injected drugs	462 (69.7%)
Drug use in past 12 months	
Any injection	326 (49.2%)
Non-injection use of only non-opioids	271 (40.9%)
Non-injection drug use of both opioids and non-opioids	390 (58.8%)
Ever injected with used needle	185 (27.9%)
Ever with unknown needle	126 (19.0%)
Frequency of injecting with others in past 12 months	
Always	81 (12.2%)
Most of the time	46 (6.9%)
Usually/sometimes/occasionally	140 (21.1%)
Never	54 (8.1%)
Other	342 (51.6%)
Location of injection drug use	
House/apartment	100 (15.1%)
Public place	563 (84.9%)
Most frequent location of injection drug use	
House/apartment	207 (31.25)
Public place	456 (68.8%)
Ever overdosed	302 (45.6%)
Overdosed in past 12 months	113 (17.0%)
Last overdose taken to ED/hospital	144 (21.7%)
**Health characteristics**	**n (%)**
Comorbid HIV	50 (7.5%)
Comorbid mental health condition (excluding substance use)	341 (51.4%)
Self-reported health status	
Excellent/very good	154 (23.2%)
Good	249 (37.6%)
Fair/poor/no answer	260 (39.2%)
Ever suicidal ideation	389 (58.7%)
Ever attempted suicide	227 (34.2%)
Attempted suicide in last 12 months	61 (9.2%)
Ever tested for Hepatitis C	562 (84.8%)
Reported last Hepatitis C test results	
Positive	258 (38.9%)
Negative	268 (40.4%)
No answer/don’t know/missing	137 (20.7%)
**Health care utilization**	**n (%)**
Received support from peer worker	277 (41.8%)
Received support from social support organization	402 (60.6%)
Have a regular doctor	373 (56.2%)
Been to a health clinic/doctor office/walk-in in past 12 months	399 (60.2%)
# of outpatient primary care visits in 1 year prior to survey completion	
Mean (SD)	9.9 (16.8)
Median (IQR)	4 (0–10)
Care engagement	
Engaged in primary care	372 (56.1%)
Not engaged in primary care	291 (43.8%)
Ever on methadone	223 (33.6%)
Currently on methadone	163 (24.6%)
Accessed addiction treatment in past 12 months	239 (36.0%)

^a^Although” Aboriginal” was the language used in the survey, we use “Indigenous” in this text to reflect current preference

^b^ADGs = Aggregated Diagnosis Groups

Table 2 compares PROUD participants who were engaged in primary care (56.1%) versus those not engaged (43.9%). Those who were engaged in primary care were more likely to be in the lowest income quintile (40.1% vs. 33.3%, *p* = 0.02 for comparison across all income strata), to be receiving disability payments (64.5% vs. 35.1%, *p* < 0.01) and to have high comorbidity (36.8% vs. 11.3%, p < 0.01 across all comorbidity strata). For social characteristics, people who were engaged in primary care were also more likely to report receiving drugs, money or gifts for sex the last 12 months (15.1% vs. 8.9%, p = 0.02), to have stable housing (45.7% vs. 26.8%, p < 0.01), and to have been red zoned (legally barred from being within a specific geographical area for a specific period of time, often due to drug-related activity in that area) (35.5% vs. 27.5%, *p* = 0.029). With respect to drug use, participants engaged in primary care were more likely to have ever injected drugs (77.7% vs. 59.5%, p < 0.01). Those engaged in primary care were also more likely to have ever taken methadone (46.2% vs. 17.5%, p < 0.01). As expected, participants engaged in primary care had many more primary care visits in the previous year (median 8 visits vs. 1 visit, p < 0.01).

**Table 2:** PROUD participant characteristics by care engagement status (non-opioid substitution therapy visits) (n = 663)

Variable	Not Engaged	Engaged	*P* value^a^
	*N* = 291	*N* = 372	
**Demographic characteristics**	n (%)	n (%)	
Age			
Mean (SD)	41.3 (10.6)	41.6 (10.9)	0.734
Median (IQR)	42 (33–50)	43 (32–50)	0.652
Age category (years)			
< = 24	26 (8.9%)	28 (7.5%)	0.406
25 to 34	56 (19.2%)	78 (21.0%)	
35 to 44	88 (30.2%)	94 (25.3%)	
45+	121 (41.6%)	172 (46.2%)	
Gender			
Male	229 (78.7%)	272 (73.1%)	0.097
Female	62 (21.3%)	100 (26.9%)	
Ethnicity			
Indigenous only	59 (20.3%)	61 (16.4%)	0.198
Other/no answer	232 (79.7%)	311 (83.6%)	
First language			
French	48 (16.5%)	54 (14.5%)	0.761
English	221 (75.9%)	291 (78.2%)	
Other/no answer	22 (7.6%)	27 (7.3%)	
Sexual orientation			
Heterosexual	263 (90.4%)	324 (87.1%)	0.188
Gay/lesbian/homosexual/other	28 (9.6%)	48 (12.9%)	
Neighborhood of residence			
Market/Lowertown	130 (44.7%)	143 (38.4%)	0.237
Centretown	46 (15.8%)	71 (19.1%)	
Other	115 (39.5%)	158 (42.5%)	
Neighborhood Income Quintile			
1 (lowest)	97 (33.3%)	149 (40.1%)	0.002
2	99 (34.0%)	97 (26.1%)	
3	62 (21.3%)	82 (22.0%)	
4 and 5 (highest)	22 (7.6%)	<=45 (0.0%)	
Missing	11 (3.8%)	<=5 (0.0%)	
Highest level of education			
Some high school or less	141 (48.5%)	167 (44.9%)	0.764
High school graduate or equivalent	84 (28.9%)	109 (29.3%)	
Some college or university	41 (14.1%)	58 (15.6%)	
College or university completed	25 (8.6%)	38 (10.2%)	
Provincial social assistance benefits			
Disability payments (Ontario Disability Support Program)	102 (35.1%)	240 (64.5%)	<.001
Income assistance (Ontario Works)	67 (23.0%)	97 (26.1%)	
Other (includes Trillium, 65y+, none)	122 (41.9%)	35 (9.4%)	
Comorbidity			
No comorbidity (0 ADGs)	77 (26.5%)	<=5 (0.0%)	<.001
Low comorbidity (1–5 ADGs)	123 (42.3%)	119 (32.0%)	
Medium comorbidity (6–9 ADGs)	58 (19.9%)	<=115 (0.0%)	
High comorbidity (> = 10 ADGs)	33 (11.3%)	137 (36.8%)	
**Social characteristics**	n (%)	n (%)	
Received drugs, money, gifts for sex last 12 months	26 (8.9%)	56 (15.1%)	0.018
Stable housing	78 (26.8%)	170 (45.7%)	<.001
Ever red zoned	80 (27.5%)	132 (35.5%)	0.029
Detained in jail overnight or longer ever	223 (76.6%)	287 (77.2%)	0.875
Detained in jail overnight or longer last 12 months	105 (36.1%)	147 (39.5%)	0.366
**Drug use characteristics**	n (%)	n (%)	
Ever injected drugs	173 (59.5%)	289 (77.7%)	<.001
Drug use in past 12 months			
Any injection	107 (36.8%)	219 (58.9%)	<.001
Non-injection use of only non-opioids	142 (48.8%)	129 (34.7%)	<.001
Non-injection drug use of both opioids and non-opioids	148 (50.9%)	242 (65.1%)	<.001
Ever injected with used needle	56 (19.2%)	129 (34.7%)	<.001
Ever with unknown needle	46 (15.8%)	80 (21.5%)	0.063
Frequency of injecting with others in past 12 months			
Always	26 (8.9%)	55 (14.8%)	<.001
Most of the time	15 (5.2%)	31 (8.3%)	
Usually/sometimes/occasionally	40 (13.7%)	100 (26.9%)	
Never	21 (7.2%)	33 (8.9%)	
Other	189 (64.9%)	153 (41.1%)	
Location of injection drug use			
House/apartment	36 (12.4%)	64 (17.2%)	0.084
Public place	255 (87.6%)	308 (82.8%)	
Most frequent location of injection drug use			
House/apartment	64 (22.0%)	143 (38.4%)	<.001
Public place	227 (78.0%)	229 (61.6%)	
Ever overdosed	114 (39.2%)	188 (50.5%)	0.004
Overdosed in past 12 months	44 (15.1%)	69 (18.5%)	0.244
Last overdose taken to ED/hospital	57 (19.6%)	87 (23.4%)	0.239
**Health characteristics**	n (%)	n (%)	
Comorbid HIV	12 (4.1%)	38 (10.2%)	0.003
Comorbid mental health condition (excluding substance use)	109 (37.5%)	253 (68.0%)	<.001
Self-reported health status			
Excellent/very good	71 (24.4%)	83 (22.3%)	0.071
Good	120 (41.2%)	129 (34.7%)	
Fair/poor/no answer	100 (34.4%)	160 (43.0%)	
Ever suicidal ideation	161 (55.3%)	228 (61.3%)	0.122
Ever attempted suicide	95 (32.6%)	132 (35.5%)	0.445
Attempted suicide in last 12 months	25 (8.6%)	36 (9.7%)	0.631
Ever tested for Hepatitis C	226 (77.7%)	336 (90.3%)	<.001
Reported last Hepatitis C test results			
Positive	78 (26.8%)	180 (48.4%)	<.001
Negative	131 (45.0%)	137 (36.8%)	
No answer/don’t know/missing	82 (28.2%)	55 (14.8%)	
**Health care utilization**	n (%)	n (%)	
Received support from peer worker	121 (41.6%)	156 (41.9%)	0.927
Received support from social support organization	179 (61.5%)	223 (59.9%)	0.682
# of outpatient primary care visits in 1 year prior to survey completion			
Median (IQR)	1 (0–3)	8 (4–19)	< 0.001
Ever on methadone	51 (17.5%)	172 (46.2%)	<.001
Currently on methadone	35 (12.0%)	128 (34.4%)	<.001
Accessed addiction treatment in past 12 months	102 (35.1%)	137 (36.8%)	0.636

^a^alpha level of 0.05 used to determine statistical significance

In our complete case adjusted analysis (*n* = 533) (Table 3), primary care engagement was associated with receiving provincial benefits, including disability payments (adjusted odds ratio [AOR] 4.14 (95% confidence interval [CI] 2.30 to 7.43)) or income assistance (AOR 3.69 (95% CI 2.00 to 6.81)). Primary care engagement was also associated with having stable housing (AOR 2.09 (95% CI 1.29 to 3.38)), having mental health comorbidity (excluding substance use disorder) (AOR 3.43 (95% CI 2.19 to 5.38)) and reporting ever taken methadone (AOR 3.82 (95% CI 2.28 to 6.41)).

**Table 3:** Multivariable logistic regression of PROUD participant characteristics associated with care engagement, excluding opioid substitution therapy visits, Complete case analysis (n = 533)

Variable	Engaged AOR^a^ (95% CI)
**Demographic characteristics**	
Age	1.00 (0.98, 1.02)
Gender	
Male	1.10 (0.63, 1.93)
Female	ref
Ethnicity	
Indigenous	1.21 (0.69, 2.10)
Other	ref
Sexual Orientation	
Heterosexual	0.78 (0.37, 1.66)
Gay/lesbian/homosexual/other	ref
Neighborhood Income quintile	
1 (Lowest)	0.55 (0.24, 1.26)
2	0.47 (0.20, 1.07)
3	0.77 (0.32, 1.82)
4 and 5 (Highest)	ref
Highest level of education	
College or university completed	0.95 (0.47, 1.93)
Some college or university	0.93 (0.49, 1.74)
High school graduate or equivalent	1.01 (0.61, 1.67)
Some high school or less	ref
Provincial social assistance benefits	
Disability payments (Ontario Disability Support Program)	4.14 (2.30, 7.43)
Income assistance (Ontario Works)	3.69 (2.00, 6.81)
Other (includes Trillium, 65y+, none)	ref
**Social characteristics**	
Received drugs, money, gifts for sex in last 12 months	
Yes	1.94 (0.85, 4.46)
Other	ref
Housing situation	
Stable housing	2.09 (1.29, 3.38)
Unstable housing	ref
Detained in jail overnight or longer in the last 12 months	
Yes	1.15 (0.72, 1.81)
Other	ref
Ever red zoned	
Yes	1.37 (0.85, 2.21)
Other	ref
**Drug use characteristics**	
Ever inject drugs	
Yes	1.01 (0.58, 1.73)
Other	ref
Overdose in the past 12 months	
Yes	0.76 (0.43, 1.36)
Other	ref
**Health characteristics**	
Comorbid HIV	
Yes	1.09 (0.45, 2.64)
No	ref
Mental health comorbidity (excluding substance use disorder)	
Yes	3.43 (2.19, 5.38)
No	ref
Reported last Hepatitis C test results	
Positive	1.20 (0.71, 2.01)
Other	ref
**Health care utilization**	
Received support from peer worker	
Yes	0.72 (0.47, 1.11)
Other	ref
Ever on methadone	
Yes	3.82 (2.28, 6.41)
Other	ref

^a^AOR = adjusted odds ratio, models adjusted for all listed covariates

In our sensitivity analysis that collapsed all “no answer”, “don’t know” and missing responses with “no”, receiving drugs, money or gifts for sex in the last 12 months had a similar effect size but the confidence interval became narrower (AOR 2.02 (95% CI 1.01 to 4.07)), and other associations from the original model persisted (Supplemental Table 1). In our sensitivity analysis that included visits exclusively for opioid substitution therapy in our categorization of engagement status (Supplemental Table 2), the same variables were associated with primary care engagement as in our primary analysis, although the effect size for each variable increased slightly.

## Discussion

Despite high comorbidity and frequent primary care visits, we found primary care engagement, defined as three or more visits to the same family physician, was low among a cohort of marginalized PWUD. We found that primary care engagement was more likely among PWUD who received provincial benefits, had stable housing, had mental health comorbidity, and reported ever being on methadone.

Considering the complexity of health care needs among PWUD, the lack of recurrent visits to any individual family physician is a concern. Our previous work has shown this cohort is also less likely to receive team-based primary care compared to the broader Ontario population [29]. Expanding on Kerr et al.’s [10] finding that 78% of PWUD self-reported visits to primary care in the past year (with a median of 8 visits [IQR 3–8]), we found that only 56% of this population had regular engagement with any one family physician (with a median of 4 visits [IQR 0–10]). While this may reflect lack of continuity with a single family physician, it also aligns with previous literature demonstrating that self-reported estimates of primary care use may be over-estimated compared to administrative sources [30–32].

Many of the characteristics we identified as associated with primary care engagement reflect the intersection of social locations and environmental risks experienced by PWUD [22], as well as the ways these social positions intersect to exacerbate their number of care needs [8, 33]. For example, people who receive disability payments, social services, and housing support likely have high mental health comorbidity [8, 34]. Despite greater reported barriers to care [7], PWUD with concomitant mental health disorders may be more likely to access medical treatment and counseling services than those with substance use disorder alone, given evidence that integration of substance use disorder services lags behind that of mental health services in primary care settings [35]. Furthermore, while unstable housing is a known barrier to accessing primary care [36], those with stable housing and who have navigated the process of applying for disability are more likely to liaise with connected or co-located health care services, including mental health and addiction services [37], with such service integration being a key policy driver for improved care [38–41]. Finally, we found that even after excluding visits for opioid substitution therapy, PWUD receiving opioid substitution therapy were more likely to be engaged in primary care. This is consistent with our previous findings that receipt of methadone was associated with an approximately 50% lowered risk of visiting an emergency department at least twice in a year [2], and likely reflects increased opportunities for care.

We used a community-based participatory research approach to gain rich survey data on a highly disadvantaged sample of PWUD. We linked this data to population-level data to characterize primary care engagement in a setting with universal health insurance. However, our study has limitations. Our survey relied on self-reported data about practices that are highly stigmatized or illegal, which may have contributed to reporting biases. We used street-based peer recruitment to improve representativeness over standard recruitment methods [21, 42]; as such, our findings may not be widely generalizable to non-street based populations. To minimize the risk of sampling bias, we recruited a large sample using focused eligibility criteria, many different recruitment locations, and with multiple steps to make participation more accessible for our marginalized target population. In addition, ICES data cannot measure all visits to nurse practitioners, nurses, or other allied health professionals, thus engagement with primary care providers other than family physicians would not be captured. However, the number of nurse practitioners in Ontario is still relatively small (22.4 per 100,000 population in 2018) compared to the number of physicians (236.5 per 100,000) [43]. Another potential limitation is that we chose one particular definition of engagement based on similar research in our region. Other definitions exist and may yield differing results. Thus, future studies may benefit from incorporating different definitions, as suitable to their objectives and health system contexts, or from incorporating sensitivity analyses comparing alternative definitions within a single study. Furthermore, as this is an observational study incorporating both survey data and health administrative data, we cannot infer a causal relationship between covariate and outcome. Our analysis of factors associated with primary care engagement may also be biased due to unmeasured confounders, such as personal beliefs about the value of health care. Similarly, the self-reported covariates were measured at only one time point. Future research intending to investigate these relationships should consider longitudinal study designs within a causal inference framework to assess the effects of changing social care and mental health care on primary care engagement. Finally, as patterns of polysubstance use are complex, distinguishing the associations between different patterns of substance use and primary care engagement was not within the scope of this study.

## Conclusions

Our study demonstrated low engagement with a regular family physician among PWUD, reflecting known unmet health care needs among this population [8]. Considering the theoretical risk environment framework, our findings highlight the prominence of the policy environment [20], consistent with previous suggestions [35], as different policies often dictate who may access methadone, disability or income assistance, and housing support, and how they do so, and also affect access to mental health specialty care. Health care systems seeking to respond to the significant morbidity and acute care use among PWUD may benefit from incorporating primary care-based models [39, 40] that emphasize improved coordination and integration of opioid substitution therapy with other medical, mental health, and substance use care [44, 45]. Such integration will require a commitment to overcome structural and philosophical barriers [46–48], with a focus on collaborative care that involves information continuity among providers, provider education, case management, and inclusion of the patient perspective [47, 49].

### Supplementary information

**Additional file 1: Table S1.** Adjusted multivariable logistic regression of PROUD participant characteristics associated with care engagement, excluding opioid substitution therapy visits. All participants (*n* = 663). Sensitivity analysis that collapsed all “no answer”, “don’t know” and missing responses with “no”.

**Additional file 2: Table S2.** Adjusted multivariable logistic regression of PROUD participant characteristics associated with care engagement, including opioid substitution therapy visits. All participants (n = 663). Sensitivity analysis that included visits exclusively for opioid substitution therapy in the categorization of engagement status.

## Data Availability

The data set from this study is held securely in coded form at ICES. While data sharing agreements prohibit ICES from making the data set publicly available, access may be granted to those who meet pre-specified criteria for confidential access, available at www.ices.on.ca/DAS. The full data set creation plan and underlying analytic code are available from the authors upon request, understanding that the programs may rely upon coding templates or macros that are unique to ICES.

## Appendix

**Table 4:** Ascertainment of mental health conditions (given the context of our cohort, we excluded substance use and alcohol use disorder which were included in the source definition) [26]

At least one hospitalization (CIHI-DAD or OMHRS) or ambulatory visit (NACRS) in the past 10 years with a diagnosis of ANY of the below diagnostic codes	
*OR*	
At least two visits in the past two years with a mental health diagnostic code (OHIP)	
Mental health diagnostic (ICD-9) codes:	
Psychotic Disorders	
295 Schizophrenia	
296 Manic-depressive psychoses, involutional melancholia	
297 Other paranoid states	
298 Other psychoses	
Non-Psychotic Disorders	
300 Anxiety neurosis, hysteria, neurasthenia, obsessive-compulsive neurosis, reactive depression	
301 Personality disorders	
302 Sexual deviations	
306 Psychosomatic illness	
309 Adjustment reaction	
311 Depressive disorder	
Social Problems	
897 Economic problems	
898 Marital difficulties	
899 Parent-child problems	
900 Problems with aged parents or in-laws	
901 Family disruption/divorce	
902 Education problems	
904 Social maladjustment	
905 Occupational problems	
906 Legal problems	
909 Other problems of social adjustment	

Ascertainment of HIV [27]

3 physician claims (International Classification of Diseases, Ninth Revision (ICD-9) code for HIV infection (042, 043, 044)) over a 3-year period (sensitivity and specificity of 96.2% (95% CI 95.2–97.9%) and 99.6% (95% CI 99.1–99.8%))

## Abbreviations

PWUD: People who use drugs
PROUD: Participatory Research in Ottawa: Understanding Drugs
OHIP: Ontario Health Insurance Plan
ADGs: Aggregated Diagnosis Groups
