# Supplementary material for: Engagement in primary health care among marginalized people who use drugs in Ottawa, Canada
Source: BMC Health Serv Res. 2020 Sep 7;20:837. doi: 10.1186/s12913-020-05670-z (PMC7487534; doi:10.1186/s12913-020-05670-z)
Supplement: Supplementary file 1 — Additional file 1: Table S1. Adjusted multivariable logistic regression of PROUD participant characteristics associated with care engagement, excluding opioid substitution therapy visits. All participants (n = 663). Sensitivity analysis that collapsed all “no answer”, “don’t know” and missing responses with “no”. [file 12913_2020_5670_MOESM1_ESM.docx]

**Supplemental Table 1**: Adjusted multivariable logistic regression of PROUD participant characteristics associated with care engagement, excluding opioid substitution therapy visits. All participants (n=663)

| Variable |  |  |  | Engaged  AOR* (95% CI) |  |
| --- | --- | --- | --- | --- | --- |
| **Demographic characteristics** |  |  |  |  | |
| Age |  |  |  | 1.00 (0.98, 1.02) | |
| Gender |  | Male |  | 1.31 (0.80, 2.14) | |
|  |  | Female |  | ref | |
| Ethnicity |  | Aboriginal |  | 1.02 (0.63, 1.65) | |
|  |  | Other/no answer |  | ref | |
| Income quintile |  | 1 (Lowest) |  | 0.64 (0.32, 1.29) | |
|  |  | 2 |  | 0.49 (0.24, 0.98) | |
|  |  | 3 |  | 0.84 (0.40, 1.78) | |
|  |  | Missing |  | 0.33 (0.06, 1.98) | |
|  |  | 4 and 5 (Highest) |  | ref | |
| Sexual Orientation |  | Heterosexual |  | 0.93 (0.50, 1.76) | |
|  |  | Gay/lesbian/homosexual/other |  | ref | |
| Highest level of education |  | College or university completed | | 1.11 (0.58, 2.11) | |
|  |  | Some college or university | | 0.99 (0.57, 1.73) | |
|  |  | High school graduate or equivalent |  | 1.00 (0.64, 1.55) | |
|  |  | Some high school or less |  | ref | |
| Provincial social assistance benefits |  | Disability payments (Ontario Disability Support Program) | | 4.64 (2.74, 7.86) | |
|  |  | Income assistance (Ontario Works) |  | 3.44 (1.99, 5.96) | |
|  |  | Other (includes Trillium, 65y+, none) |  | ref | |
| **Social characteristics** |  |  |  |  | |
| Received drugs, money, gifts for sex in last 12 months | | Yes |  | 2.02 (1.01, 4.07) | |
|  |  | Other |  | ref | |
| Housing situation |  | Stable housing |  | 2.08 (1.36, 3.17) | |
|  |  | Unstable housing |  | ref | |
| Detained in jail overnight or longer in the last 12 months | | Yes |  | 1.25 (0.83, 1.88) | |
|  |  | Other |  | ref | |
| Ever red zoned |  | Yes |  | 1.44 (0.96, 2.18) | |
|  |  | Other |  | ref | |
| **Drug use characteristics** |  |  |  |  | |
| Ever inject drugs |  | Yes |  | 1.21 (0.75, 1.93) | |
|  |  | Other |  | ref | |
| Overdose in the past 12 months |  | Yes |  | 0.83 (0.49, 1.38) | |
|  |  | Other |  | ref | |
| **Health characteristics** |  |  |  |  | |
| HIV positive at survey date |  | Yes |  | 1.88 (0.86, 4.12) | |
|  |  | No |  | ref | |
| Mental health comorbidity (excluding substance use disorder) |  | Yes |  | 2.61 (1.75, 3.89) | |
|  |  | No |  | ref | |
| Last Hepatitis C test positive |  | Yes |  | 1.37 (0.87, 2.17) | |
|  |  | Other |  | ref | |
| **Health care utilization** |  |  |  |  | |
| Received support from peer worker |  | Yes |  | 0.68 (0.46, 1.00) | |
|  |  | Other |  | ref | |
| Ever on methadone |  | Yes |  | 3.00 (1.89, 4.76) | |
|  |  | Other |  | ref | |
|  |  |  |  |  | |
| *AOR = adjusted odds ratio |  |  |  |  | |
|  |  |  |  |  | |
|  |  |  |  |  | |
|  | | | | | |
