# Supplementary material for: Engagement in primary health care among marginalized people who use drugs in Ottawa, Canada
Source: BMC Health Serv Res. 2020 Sep 7;20:837. doi: 10.1186/s12913-020-05670-z (PMC7487534; doi:10.1186/s12913-020-05670-z)
Supplement: Supplementary file 2 — Additional file 2: Table S2. Adjusted multivariable logistic regression of PROUD participant characteristics associated with care engagement, including opioid substitution therapy visits. All participants (n = 663). Sensitivity analysis that included visits exclusively for opioid substitution therapy in the categorization of engagement status. [file 12913_2020_5670_MOESM2_ESM.docx]

**Supplemental Table 2:** Adjusted multivariable logistic regression of PROUD participant characteristics associated with care engagement, including opioid substitution therapy visits. All participants (n=663)

| Variable | |  | |  |  | Engaged  AOR* (95% CI) |
| --- | --- | --- | --- | --- | --- | --- |
| Demographic characteristics | | | | |  |  |
| Age |  | |  | |  | 1.00 (0.98, 1.02) |
| Gender |  | | Male | |  | 1.28 (0.74, 2.20) |
|  |  | | Female | |  | ref |
| Ethnicity |  | | Aboriginal | |  | 0.86 (0.52, 1.43) |
|  |  | | Other/no answer | |  | ref |
| Income quintile |  | | 1 (Lowest) | |  | 0.70 (0.33, 1.50) |
|  |  | | 2 | |  | 0.49 (0.23, 1.06) |
|  |  | | 3 | |  | 0.95 (0.42, 2.13) |
|  |  | | Missing | |  | 0.26 (0.04, 1.76) |
|  |  | | 4 and 5 (Highest) | |  | ref |
| Sexual Orientation |  | | Heterosexual | |  | 0.73 (0.36, 1.46) |
|  |  | | Gay/lesbian/homosexual/other | |  | ref |
| Highest level of education | | | College or university completed | | | 1.32 (0.66, 2.65) |
|  |  | | Some college or university | | | 1.05 (0.57, 1.93) |
|  |  | | High school graduate or equivalent | |  | 1.10 (0.68, 1.77) |
|  |  | | Some high school or less | |  | ref |
| Provincial social assistance benefits | | | Disability payments (Ontario Disability Support Program) | | | 5.78 (3.30, 10.12) |
|  |  | | Income assistance (Ontario Works) | |  | 4.53 (2.52, 8.15) |
|  |  | | Other (includes Trillium, 65y+, none) | |  | ref |
| **Social characteristics** |  | |  | |  |  |
| Received drugs, money, gifts for sex in last 12 months | | | Yes | |  | 2.26 (1.04, 4.93) |
|  |  | | Other | |  | ref |
| Housing situation |  | | Stable housing | |  | 2.73 (1.72, 4.34) |
|  |  | | Unstable housing | |  | ref |
| Detained in jail overnight or longer in the last 12 months | | | Yes | |  | 1.36 (0.87, 2.13) |
|  |  | | Other | |  | ref |
| Ever red zoned |  | | Yes | |  | 1.34 (0.86, 2.09) |
|  |  | | Other | |  | ref |
| **Drug use characteristics** |  | |  | |  |  |
| Ever inject drugs |  | | Yes | |  | 1.14 (0.70, 1.88) |
|  |  | | Other | |  | ref |
| Overdose in the past 12 months | | | Yes | |  | 0.93 (0.53, 1.63) |
|  |  | | Other | |  | ref |
| **Health characteristics** |  | |  | |  |  |
| HIV positive at survey date | | | Yes | |  | 1.65 (0.70, 3.87) |
|  |  | | No | |  | ref |
| Mental health comorbidity (excluding substance use disorder) | | | Yes | |  | 2.91 (1.89, 4.50) |
|  |  |  | No | |  | ref |
| Last Hepatitis C test positive | | | Yes | |  | 1.52 (0.92, 2.51) |
|  |  | | Other | |  | ref |
| **Health care utilization** |  | |  | |  |  |
| Received support from peer worker | | | Yes | |  | 0.72 (0.47, 1.09) |
|  |  | | Other | |  | ref |
| Ever on methadone |  | | Yes | |  | 6.29 (3.64, 10.87) |
|  |  | | Other | |  | ref |
|  | |  | |  |  |  |
| *AOR = adjusted odds ratio | | | |  |  |  |
